# Renal capsule for augmentation cystoplasty in canine model: a favorable biomaterial?

**DOI:** 10.1590/S1677-5538.IBJU.2014.0680

**Published:** 2016

**Authors:** Mehdi Salehipour, Reza Mohammadian, Amir Malekahmadi, Massood Hosseinzadeh, Mahnaz Yadollahi, Mohammad Natami, Mahsa Mohammadian

**Affiliations:** 1 Department of Urology, Shiraz University of Medical Sciences, Shiraz, Iran; 2 Department of Pathology, Shiraz University of Medical Sciences, Shiraz, Iran; 3 Trauma Research Center, Shiraz University of Medical Sciences, Shiraz, Iran

**Keywords:** Urinary Bladder, Dogs, Biocompatible Materials

## Abstract

**Purpose:**

To evaluate effectiveness of canine renal capsule for augmentation cystoplasty.

**Materials and Methods:**

Ten adult dogs participated in this study. After induction of anesthesia each animal underwent bed side urodynamic study, bladder capacity and bladder pressure was recorded. Then via mid line incision abdominal cavity was entered, right kidney was identified and its capsule was dissected. Bladder augmentation was done by anastomosing the renal capsule to the bladder. After 6 months bed side urodynamic study was performed again and changes in bladder volume and pressure were recorded. Then the animals were sacrificed and the augmented bladders were sent for histopathology evaluation.

**Results:**

Mean maximum anatomic bladder capacity before cystoplasty was 334.00±11.40cc which increased to 488.00±14.83cc post-operatively (p=0.039). Mean anatomic bladder pressure before cystoplasty was 19.00±1.58cmH2O which decreased to 12.60±1.14cmH_2_O post-operatively (p=0.039). Histopathology evaluation revealed epithelialization of the renal capsule with urothelium without evidence of fibrosis, collagen deposits or contracture.

**Conclusions:**

Our data shows that renal capsule is a favorable biomaterial for bladder augmentation in a canine model.

## INTRODUCTION

Augmentation cystoplasty (AC) aims to change the high pressure low capacity bladder into a stable environment for urine storage and emptying, so that it would prevent renal function deterioration and preserve continence. It was first described in the canine model by Tizzoni and Foggi in 1888 (1) and later in humans by von Mikulicz in 1889 (2). Since the introduction of AC, bowel segments have been used in different studies and have become a relatively favorable tissue for bladder augmentation, however, using bowel segment for AC is a complex procedure and its untoward complications such as metabolic disorders, urinary tract infections (UTI), stone formation and malignancy have been mentioned in different series (3–6). In order to find an ideal replacement for bowel segments different studies testing different natural or synthetic tissues have been conducted most of which have not been promising (7–9). Renal capsule is a thin sheet of connective tissue containing blood vessels, fibroblast and adipose tissue with a good tensile strength. In an attempt to introduce a new biomaterial as a scaffold for urothelial regeneration, we used the renal capsule for bladder augmentation in a canine model.

## MATERIALS AND METHODS

**Figure 1:**
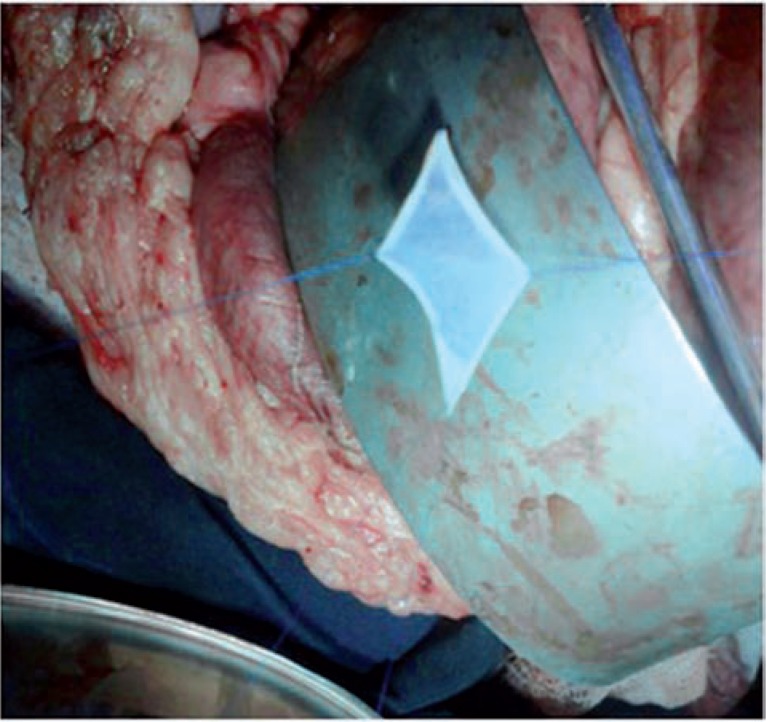
preparing the renal capsule.

**Figure 2:**
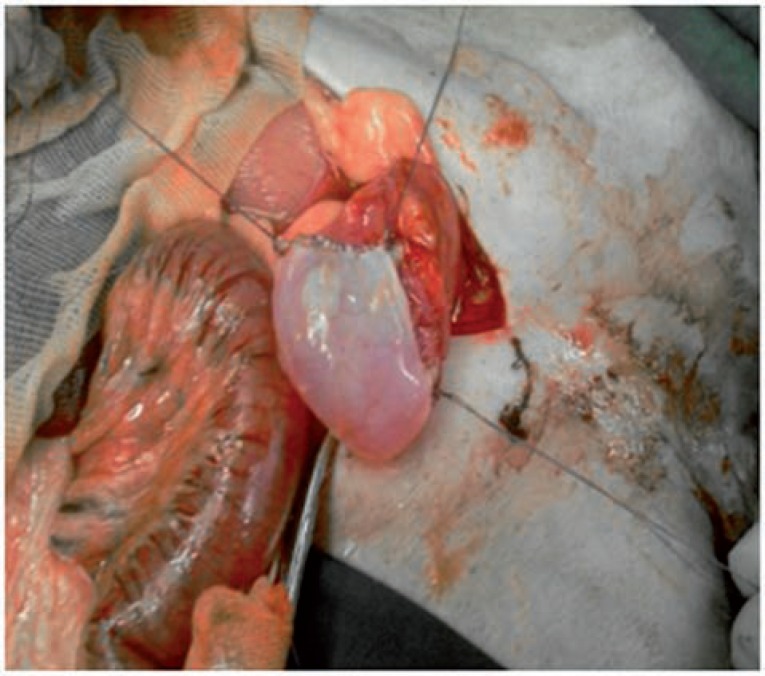
Anastomosing the renal capsule to the bladder (bladder augmentation).

**figure 3:**
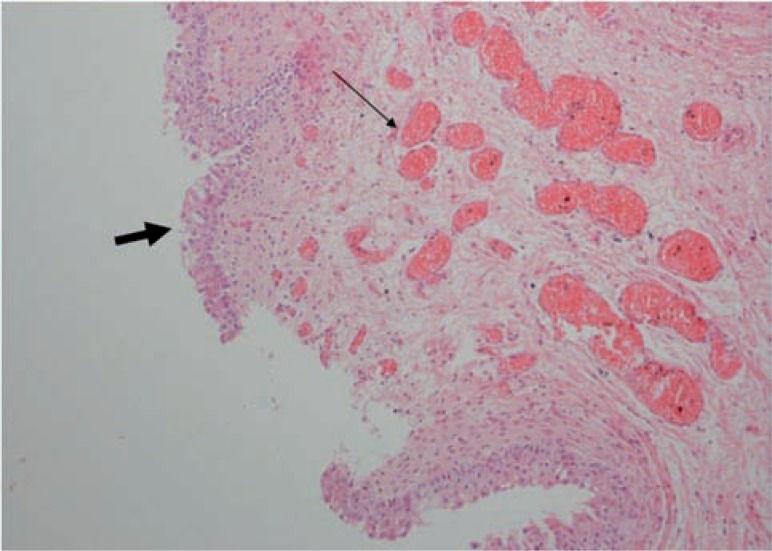
The microscopic examination show surface urothelial epithelialization (thick arrow) and neovascularization of stroma (thin arrow), H&E X 100.

**Figure 4:**
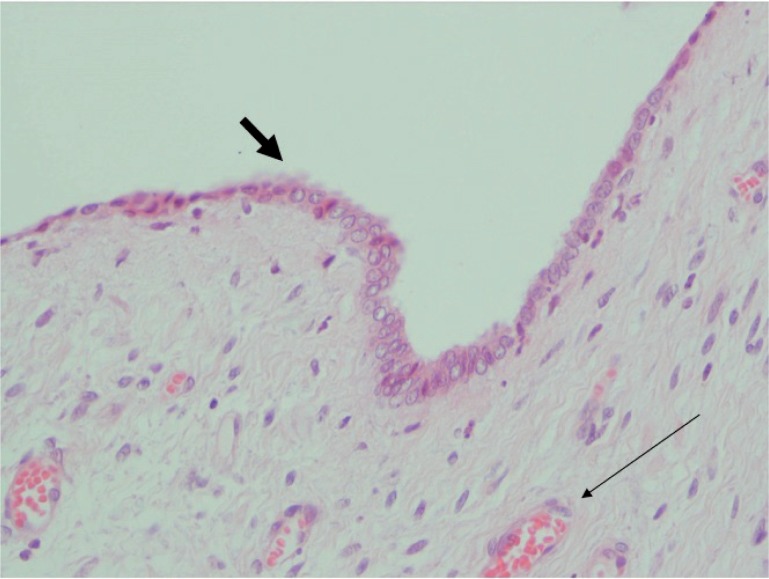
The thick arrow shows surface epithelialization and thin arrow reveals neovascularization, H&E x 400.

**Figure 5:**
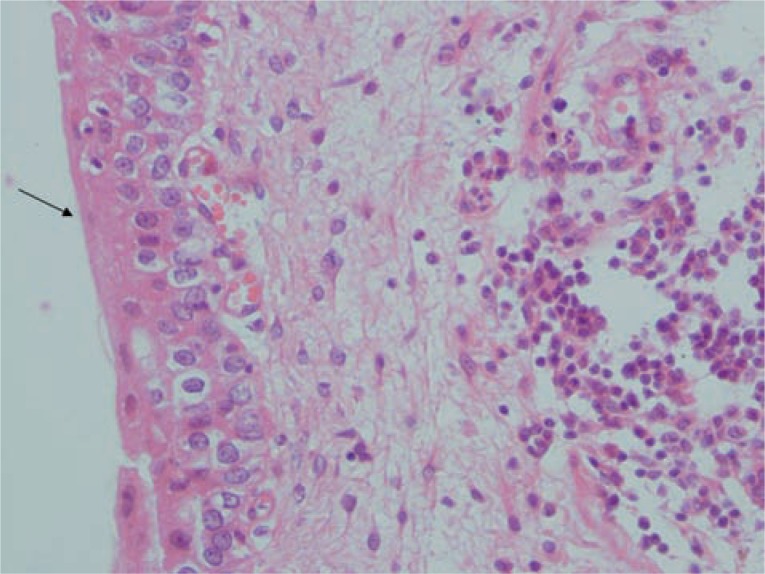
The arrow shows surface epithelialization, H&E x 400.

This experimental study was carried out on ten mixed breed adult dogs (20±2 months) weighing about 15kg. The protocol was approved by the Institutional Animal Care and Medical Ethics Committee of our center in accordance with the principles of laboratory animal care formulated by the national society of medical research and the guidelines for the care and use of laboratory animals published by the National Institute of Health (1996) (10). All the animals were allowed a minimum of 72 hours to recover from the stress of transportation before the first procedure and food was withheld for 12 hours before anesthesia. Intramuscular ampicillin (100mg/kg), gentamicin (5mg/kg), and clindamycin (20mg/kg) were injected 15 minutes before starting the operation, all interventions were performed in a standard operating room. General anesthesia was induced using intravenous ketamine hydrochloride (3mg/kg) and diazepam (0.3mg/kg). After intubating each animal, anesthesia was maintained with inhaled halothane with the depth monitored to maintain an absent withdrawal reflex to toe pinch and absent corneal reflex. After induction of anesthesia, each animal underwent eyeball urodynamic study according to the standard protocol (11, 12). Each dog was placed in the supine position and catheterized with a 10Fr catheter. A 50-60mL catheter tip syringe was chosen, the barrel of the syringe was connected directly to the end of the catheter and it was held upright at the level of symphysis pubis. Saline was infused into the bladder by pouring into the open end of the syringe. The height of the barrel is raised or lowered until there is steady flow. As the saline flow stopped, the height of the meniscus, above the symphysis, was considered the measured vesical pressure. The amount of fluid flew in to the bladder was considered the measure of bladder volume and was defined as the maximum anesthetic bladder capacity (MABC) and the pressure at MABC was defined as the maximum anesthetic bladder pressure (MABP) (11). Then through a mid line abdominal incision, the right kidney was identified. A circumferential incision was made over the renal hilum and the renal capsule was meticulously dissected from the underlying parenchyma (Figure-1). After preparing the renal capsule (5x10cm), cystotomy was done through a vertical incision on the bladder dome and subsequently the capsule was anastomosed to the bladder using polydioxanone 4-0 suture (Figure-2). A 10Fr internal Foley catheter was inserted into the bladder, the wound was closed and an appropriate dressing was applied. Antibiotic therapy with ampicillin (300mg q 6h) was continued for one week after the procedure and the internal Foley catheter was removed at the end of the first week after the operation. During the recovery period (third and seventh day after the operation) we asked the caretaker of the animal laboratory to keep each animal calm so we could perform a brief physical examination of the operation site for detection of hematoma or infection. Six months after the operation, under general anesthesia, bed side urodynamic study was performed for each animal again and the changes in urodynamic parameters were documented; then the animals were sacrificed sign of hematoma or infection. During the exploration in the sixth month, no gross abnormality of the decapsulized kidneys was detected. The preoperative mean MABC was 334.00±11.40mL, which increased to 488.00±14.83mL (p=0.03) after the operation. Preoperative mean MABP was 19.00±1.58cmH_2_O, which decreased to 12.60±1.14cmH_2_O postoperatively (p=0.03) (Table-1). In histopathology evaluation all the sections showed well formed granulation tissue containing neovascularization and fibroblast proliferation (Figures 3 and 4). In six of ten samples (60%) there was full urothelial epithelialization (Figure-5). The rest of tissue other than that with full epithelialization showed repair by connective tissue rather than urothelial proliferation. Indeed the microscopic examination in other 40% of samples revealed fibroblast proliferation, angiogenesis and collagen deposition. Evidence of urothelial proliferation in 60% of samples on HεtE slides were and the augmented bladders were removed and sent for histopathology evaluation. All cystectomy specimens were sent for histopathology evaluation after fixation in 10% buffered formalin. Macroscopic examination performed and then five sections were prepared from each specimen. After tissue processing and paraffin block preparation, 5μm sections were inserted on glass slides. The slides were stained with Hematoxylin and Eosin (H&E) method and examined under light microscopy.

**Table 1:** Results of urodynamic evaluation before and after operation.

	Before Operation	After Operation	P-value
mean MABC (mL)	334.00±11.40	488.00±14.83	0.03
mean MABP (cm H_2_O)	19.00±1.58	12.60±1.14	0.03
Number of Animals	10	10	

## RESULTS

Ten mixed breed adult dogs were enrolled in this study. During the recovery period physical examination of the operation site did not show any stratification of epithelial cells with abundant amphophilic to clear cytoplasms and coniform superficial borders. Moreover, we applied immunohistochemistry against cytokeratin 7 for the confirmation of transitional epithelium. There was no evidence of necrosis, fibrosis, acute inflammation or ulceration in any cystectomy specimen.

## DISCUSSION

Congenital or acquired disorders of the urinary bladder can make it a non–compliant environment for storage and emptying of urine, causing complications such as recurrent UTIs, irritative urinary symptoms, urinary incontinence, vesicoureteral reflux, or even chronic renal failure. To overcome this problem, AC was first described in the canine model by Tizzoni and Foggi in 1888 (1) and later in humans by von Mikulicz in 1889 (2). Since that time finding an ideal tissue for augmenting the bladder has been debated, however, bowel segments and particularly ileum still are the gold standard tissues for AC. However, apart from its complexity, augmenting the bladder with gastrointestinal segment has some problematic outcomes described by different studies such as UTI, bladder stones, hematuria - dysuria syndrome, metabolic derangements, and malignancy especially at the uretero-intestinal anastomoses (3–6). Moreover, the use of bowel segment will be limited or contraindicated in circumstances including short bowel syndrome, inflammatory bowel disease, congenital anomalies such as cloacal exstrophy or after radiotherapy (13). Auto––augmentation or detrusor myomectomy was first described in 1989 (14) and aimed to create a low pressure bladder by resecting the detrusor off the bladder and creating a diverticulum. Although this technique can decrease the bladder pressure and improve its capacity and compliance, there may be increased risk of incomplete emptying requiring clean intermittent catheterization (15). Auto-augmentation is simple and associated with less morbidity when compared with enterocystoplasty with the overall success rate of 70% for idiopathic detrusor overactivity and 50% for neurogenic detrusor overactivity, however, the long term results should be evaluated further (16). Ureterocystoplasty uses a dilated ureter for bladder augmentation by forming it to a patch. Since it is the urothelium that augments the bladder the complications would be much less when compared to enterocystoplasty and long term follow-up showed acceptable improvements in bladder capacity and compliance (17). Unfortunately the use of this technique is limited to those with megaureters or patients with non-functioning native kidneys and hydrouereteronephrosis. Natural tissues including free fascial grafts, omentum, peritoneum, dura matter and placenta used for AC were unsuccessful with a high rate of complications (7, 8). Synthetic materials such as teflon, polyvinyl sponge, gelatin sponge and resin coated papers have all been used for prosthetic bladder augmentation but all proved to be useless due to problems including recurrent UTIs, stone formation, metaplastic reactions, contracture and fibrosis (8, 9). Renal capsule is a thin layer of connective tissue which is composed of blood vessels, fibroblast and adipose tissue. This structure has a good tensile strength with a low metabolic rate and minimal fluctuation in the physic-mechanical parameters which makes it a suitable protector of the renal parenchyma (18) and most likely a favorable natural tissue for reconstructive procedures. Thompson et al. reported their clinical experience of flap pyeloplasty using renal capsule and their results were acceptable (19). Hodjati et al. (20) applied the canine renal capsule as a venous patch graft for inferior vena cava repair and their entire specimens remained patent with endothelial regeneration over the surface of renal capsule 3 months after the operation. To the author's knowledge our study is the first experience of AC using renal capsule, which we introduce as capsule-cystoplasty. Recent study showed that all the canine bladders that were augmented with renal capsule had a significant increase in capacity with a significant decreased in pressure, proven urodynamically. Histopathology evaluation revealed epithelialization of the capsule with urothelium and the amazing issue to be considered is that in none of the specimens, fibrosis, graft shrinkage or metaplastic reaction was observed. The recent finding might be attributed to the low metabolic rate of the renal capsule which necessitates a low blood flow with subsequent less aggregation of inflammatory cells, and also it's more compatible histology to the urinary tract.

Recently application of biodegradable materials for AC has gained interest because of their ease of usage and lower complication rates compared to the bowel segments, however, some limitations still exist. Paterson et al. (21) and Wang et al. (22) conducted separate studies comparing autogenous ileum with small intestinal submucosa (SIS) for laparoscopic bladder augmentation in porcine models and both studies yielded better results with ileum. Their histopathological evaluation revealed more intra-abdominal fibrosis and also contracture, scar formation, and metaplastic bone formation in the walls of bladders augmented with SIS which could explain the lower bladder dynamics resulted in SIS group. In our study, we didn't detect any fibrosis and scar formation in the bladders augmented with renal capsule which shows its priority to SIS, however, a comparative study evaluating the urodynamic parameters after enterocystoplasty versus capsule-cystoplasty would be of value. The feasibility of renal capsule for AC in human has not been proved yet. One can easily use the renal capsule for AC in patients with non-functional native kidneys without the fear of damaging the renal parenchyma. Although Hodjati et al. (20) showed no renal parenchymal damage after removing the capsule in canine models, and we also did not find any gross abnormality of the decapsulized kidneys during second exploration, there is still concern for its applicability in humans with normally functioning kidneys.

## CONCLUSIONS

According to our clinical and pathological data renal capsule may act as a suitable biomaterial for bladder augmentation in a canine model, however, we recommend a case-control study with more participants to validate our results. Besides, the applicability of renal capsule in human should be verified.

## Abbreviations

AC: = Augmentation Cystoplasty
UTI: = Urinary Tract Infection
MABC: = Maximum Anatomic Bladder Capacity
MABP: = Maximum Anatomic Bladder Pressure
H&E: = Hematoxylin and Eosin
